# Complete Genome Sequence of *Bradyrhizobium japonicum* J5, Isolated from a Soybean Nodule in Hokkaido, Japan

**DOI:** 10.1128/genomeA.01619-16

**Published:** 2017-02-09

**Authors:** Kazuma Kanehara, Kiwamu Minamisawa

**Affiliations:** Graduate School of Life Sciences, Tohoku University, Sendai, Japan

## Abstract

Soybean bradyrhizobia form root nodules on soybean plants and symbiotically fix N_2_. Strain J5 is phylogenetically far from well-known representatives within the *Bradyrhizobium japonicum* linage. The complete genome showed the largest single chromosomal (10.1 Mb) and symbiosis island (998 kb) among complete genomes of soybean bradyrhizobia.

## GENOME ANNOUNCEMENT

Soybean bradyrhizobia are symbiotic nitrogen-fixing bacteria with soybean plants (1) and are composed of two major species, *Bradyrhizobium diazoefficiens* and *B. japonicum*, in temperate areas (2). The complete genomes of *B. diazoefficiens* USDA110 (3), *B. diazoefficiens* NK6 (4), *B. japonicum* USDA6 (5), and *B. japonicum* E109 (6) revealed their dynamic structures, including a symbiosis island and insertion sequence–mediated rearrangements (4–7). Strain J5 is phylogenetically far from USDA6 and E109 within the diverse *B. japonicum* linage. Thus, we determined the complete genome sequence of *B. japonicum* J5.

Strain J5 was isolated by Toshikazu Takahashi (Tokachi Federation of Agricultural Cooperative Associations) from the nodule of a soybean plant grown in Shibetsu City in Hokkaido, Japan, in 1998. The genome was sequenced using the PacBio RSII (Pacific Biosciences, Menlo Park, CA USA) and Illumina MiSeq (Illumina, San Diego, CA, USA) platforms. Genomic DNA libraries were prepared for PacBio sequencing using the SMRTbell template prep kit version 1.0, while Illumina libraries were prepared using the Nextera DNA sample preparation kit (Illumina). The resulting reads, generated from the PacBio RSII sequencing procedure, were assembled *de novo* using the Hierarchical Genome Assembly Process (HGAP3.0) approach (SMRT analysis version 2.3.0) with default parameters. The final draft assembly produced two contigs totaling 10.3 Mb in size and with an input read coverage of 28×. Row reads generated from the Illumina MiSeq sequencing procedure were trimmed using CLC Genomics Workbench version 8.5.1 (CLC Bio, Aarhus, Denmark) with the following parameters: ambiguous limit, 2; quality limit, 0.05; number of 5′ terminal nucleotides, 20; number of 3′ terminal nucleotides, 5; and minimum number of nucleotides in reads, 70. Trimmed whole-genome shotgun reads were mapped to contigs to update them, with the following CLC Genomics Workbench parameters: mismatch cost, 2; insertion cost, 3; deletion cost, 3; length fraction, 0.5; similarity fraction, 0.8; and no masking. As a result, a single circular contig was assembled. The sequence was annotated using the NCBI Prokaryotic Genome Automatic Annotation Pipeline (8), and the result was manually inspected with respect to positions of start codons for predicted open reading frames using the Microbial Genome Annotation Pipeline (MiGAP; http://www.migap.org) and GenomeMatcher (9).

The J5 genome consisted of a single chromosome (10,138,651bp, 63.3% G+C) and 9,067 coding sequences (CDSs). The genome size and CDS numbers were the highest among complete genomes of soybean bradyrhizobia. The length of symbiosis island A (5) reached 998 kb, which is the largest symbiosis island in soybean bradyrhizobia (643 kb to 694 kb). Genome-to-genome distances (high-scoring segment pair length/total length) were calculated using GGDC version 2.1 (http://ggdc.dsmz.de/distcalc2.php) (10). The distance between *B. japonicum* strain J5 and strains USDA6 and E109 (0.0441 to 0.0439) was apparently larger than that between USDA6 and E109 (0.0003), indicating that J5 was far from the well-known representatives within the *B. japonicum* linage in terms of genomics as well. As for denitrification genes relevant to global warming (11), the J5 genome lacked the *nos* gene cluster encoding N_2_O reductase.

### Accession number(s).

The genome sequence of *B. japonicum* strain J5 has been deposited at the GenBank under the accession number CP017637.
